# Integrating Technology into Urticaria Management: Telemedicine, Remote Monitoring and Patient-Centered Care

**DOI:** 10.3390/biomedicines14040753

**Published:** 2026-03-26

**Authors:** Ester Topa, Mattia Cristallo, Angela Rizzi, Donatella Lamacchia, Sara Gamberale, Cristiano Caruso, Oliviero Rossi, Elisabetta Di Leo, Maria Bova, Eustachio Nettis

**Affiliations:** 1 Division of Internal Medicine 2, Department of Medicine and Medical Specialties, A. Cardarelli Hospital, 80131 Naples, Italy; topaester@gmail.com (E.T.); bovamaria@virgilio.it (M.B.); 2 Department of Precision and Regenerative Medicine and Ionian Area, Regional Reference Center for Allergic and Immunological Diseases, University of Bari Aldo Moro, 70124 Bari, Italy; cristallomattia@gmail.com (M.C.); ambulatorio.allergologia@uniba.it (E.N.); 3 UOSD Allergy and Clinical Immunology Unit, Fondazione Policlinico A. Gemelli IRCCS, 00168 Rome, Italy; gamberalesara@gmail.com (S.G.); carusocristiano1@gmail.com (C.C.); 4 Personalized Medicine, Asthma and Allergy, Humanitas Clinical and Research Center IRCCS, Via Alessandro Manzoni 56, 20089 Rozzano, Italy; donatella.lamacchia@humanitas.it; 5 Facoltà di Medicina e Chirurgia, Università Cattolica del Sacro Cuore, Largo Francesco Vito 1, 00168 Rome, Italy; 6 Postgraduate School of Allergy and Clinical Immunology, University of Florence, 50134 Florence, Italy; oliviero.rossi@unifi.it; 7 Section of Allergy and Clinical Immunology, Unit of Internal Medicine, “F. Miulli” Hospital, Strada Provinciale per Santeramo Km 4.100, 70021 Acquaviva delle Fonti, Italy; elisabettadileo71@libero.it

**Keywords:** urticaria, chronic urticaria, telemedicine, digital health, patient-reported outcomes, quality of life, disease monitoring, personalized medicine, dermatology, allergy

## Abstract

**Background**: Urticaria, particularly chronic urticaria (CU), is a highly prevalent inflammatory skin disorder characterized by recurrent wheals and/or angioedema with a fluctuating and unpredictable course that significantly impairs quality of life and requires long-term monitoring. Despite established therapeutic guidelines, disease control remains suboptimal in a considerable proportion of patients. Telemedicine has emerged as a promising adjunctive strategy for chronic disease management. This review aims to critically evaluate the role, applications, benefits, and limitations of telemedicine and digital health interventions in urticaria management. **Methods**: A scoping review of the literature was conducted focusing on studies addressing telemedicine, digital patient-reported outcomes, mobile health applications, and remote monitoring strategies in urticaria. Evidence from pandemic and post-pandemic telemedicine models was also analyzed to identify transferable approaches. **Results**: Telemedicine demonstrates significant potential in urticaria management by enabling structured symptom monitoring, facilitating remote follow-up during therapeutic escalation (including biologic therapies), improving patient empowerment and adherence, and reducing healthcare utilization and indirect costs. Digital tools such as electronic diaries and validated PRO-based applications support continuous disease assessment. However, telemedicine cannot replace direct clinical examination, and limitations include diagnostic uncertainty, digital inequalities, data privacy concerns, and lack of large disease specific trials. **Conclusions**: Telemedicine represents a valuable complementary and integrative model for urticaria care, particularly suited for chronic disease monitoring. Hybrid care pathways combining remote and in-person management appear to be the most effective and sustainable strategy. Further high-quality urticaria-specific studies and standardized digital frameworks are required to optimize its clinical implementation.

## 1. Introduction

Urticaria is a frequent inflammatory skin disorder characterized by recurrent appearance of transient wheals, angioedema, or both, resulting from localized plasma extravasation in the superficial or deep dermis. Lesions are typically accompanied by intense pruritus or burning sensations and resolve spontaneously within 24 h, although new lesions may continuously emerge. Based on disease duration, urticaria is classified as acute, when symptoms resolve within six weeks, or chronic, when wheals and/or angioedema persist or recur for more than six weeks [1]. Chronic urticaria (CU) encompasses two main entities: chronic spontaneous urticaria (CSU), which occurs in the absence of identifiable external triggers, and chronic inducible urticaria (CIndU), in which symptoms are reproducibly elicited by specific physical or environmental stimuli, including cold, heat, pressure, vibration, or ultraviolet radiation [1].

From an epidemiological perspective, urticaria represents a substantial global health burden. Acute urticaria affects up to one in five individuals at least once during their lifetime, whereas chronic urticaria has a point prevalence ranging between 0.5% and 1% worldwide [1,2]. CU may affect individuals of all ages but shows a higher prevalence among adults and a female predominance. Although rarely life-threatening, CU is often persistent, with a disease duration extending over several years in a significant proportion of patients and is associated with frequent relapses and variable disease activity [3].

The pathophysiology of urticaria is complex and multifactorial [4], involving dysregulated activation of mast cells and basophils as central effector cells. Upon activation, these cells release histamine and other pro-inflammatory mediators, including cytokines, proteases, and lipid-derived mediators, which increase vascular permeability and stimulate sensory nerve endings, leading to wheal formation, erythema, pruritus, and angioedema [5]. In chronic urticaria, increasing evidence supports the involvement of autoimmune mechanisms, including IgG autoantibodies directed against the high-affinity IgE receptor (FcεRI) or IgE itself, as well as IgE autoantibodies targeting self-antigens, which may contribute to sustained mast cell activation and disease chronicity [6,7]. Neuroimmune interactions and coagulation pathway activation have also been implicated in disease pathogenesis, further highlighting the heterogeneity of underlying mechanisms.

Clinically, urticaria presents with intensely pruritic wheals of variable size and shape, often surrounded by erythema and accompanied by angioedema in a subset of patients. Angioedema may involve the lips, eyelids, extremities, or mucosal tissues and is frequently associated with pain or discomfort rather than itching. Symptoms are often unpredictable, fluctuate over time, and may be exacerbated by physical, emotional, or environmental factors, including stress and infections. This variability complicates disease assessment and contributes to the substantial burden experienced by patients.

Current therapeutic strategies are primarily aimed at achieving complete symptom control and improving patients’ quality of life. According to international guidelines, second-generation H1-antihistamines are recommended as first-line therapy, with dose escalation up to fourfold in patients with inadequate response [1]. For patients with antihistamine-refractory disease, biologic therapies, particularly anti-IgE monoclonal antibodies, and immunomodulatory agents are recommended as subsequent treatment options [8,9]. Despite the availability of effective therapies and evidence-based guidelines, disease control remains suboptimal in a considerable proportion of patients, largely due to the chronic relapsing nature of urticaria, interindividual variability in treatment response, and the need for long-term, personalized management.

In recent years, telemedicine and digital health solutions have emerged as important supportive tools in the management of chronic inflammatory diseases, including chronic urticaria. The clinical characteristics of urticaria—such as fluctuating disease activity, the need for frequent follow-up, continuous monitoring of treatment response, and systematic identification of potential triggers—make this condition particularly well suited to remote care models [10]. Teleconsultations, mobile health applications, and digital platforms enable regular follow-up without the logistical constraints of in-person visits, facilitating timely assessment of disease control and early identification of symptoms worsening. In addition, the use of digital symptom diaries allows for continuous and standardized evaluation of disease severity over time [7,10]. Collectively, these approaches support timely therapeutic adjustments, enhance continuity of care, and may reduce the burden of face-to-face consultations, positioning telemedicine as a promising adjunct to traditional care models with the potential to improve patient engagement, adherence to therapy, and overall disease control [11].

Monitoring potential triggers represents another key aspect of urticaria management that may benefit from digital solutions. Mobile health applications enable patients to systematically record environmental exposures, lifestyle factors, and symptom patterns, supporting both patients and clinicians in identifying relevant triggers and tailoring management strategies accordingly. Moreover, teleconsultations can enhance patient–physician communication, promote shared decision-making, and support treatment adherence through ongoing education and feedback [11].

From a healthcare system perspective, telemedicine may contribute to improved resource utilization by reducing unnecessary outpatient visits and facilitating earlier therapeutic adjustments, particularly in patients with stable disease or those requiring close monitoring during treatment escalation. By empowering patients and enabling continuous engagement, digital health interventions have the potential to improve clinical outcomes, enhance quality of life, and reduce the overall burden associated with chronic urticaria.

## 2. Burden of Disease and Patient Experience

Chronic urticaria is associated with a substantial and multifaceted disease burden that affects patients’ physical, psychological, and social well-being. Persistent pruritus, recurrent wheals, and unpredictable episodes of angioedema significantly impair health-related quality of life, often to an extent comparable to other chronic inflammatory skin diseases such as psoriasis and atopic dermatitis [12,13]. Sleep disturbance is among the most frequently reported symptoms and contributes to fatigue, impaired daytime functioning, and reduced productivity [14]. The visible and recurrent nature of skin lesions may further lead to social embarrassment, stigmatization, and avoidance behaviors, negatively impacting interpersonal relationships and daily activities.

In addition to its physical manifestations, chronic urticaria exerts a pronounced psychological burden. High rates of anxiety and depressive symptoms have been consistently reported, largely driven by the unpredictable disease course, fear of sudden exacerbations, and uncertainty surrounding trigger identification and treatment effectiveness [13,15]. Patients often describe a persistent perception of limited disease control, which adversely affects coping strategies, treatment satisfaction, and overall engagement with care.

From an economic perspective, chronic urticaria imposes a substantial burden on both healthcare systems and society. Direct medical costs arise from repeated outpatient visits, diagnostic evaluations, and long-term pharmacological treatments, particularly in patients with moderate-to-severe disease requiring advanced therapies such as biologics [3,16]. Given the chronic, relapsing, and often unpredictable course of the disease, patients frequently require ongoing clinical monitoring and therapy adjustments, resulting in cumulative healthcare expenditures over time.

Beyond direct healthcare costs, chronic urticaria is associated with considerable indirect costs, including absenteeism, presenteeism, reduced work productivity, and limitations in daily activities. These indirect costs account for a substantial proportion of the overall economic impact of the disease and, in some studies, exceed direct medical costs [11,12,13]. The fluctuating severity of symptoms, sleep disturbances, and the psychological burden associated with chronic urticaria further exacerbate productivity loss and social costs.

In this context, telemedicine and digital health interventions offer promising opportunities to address several unmet economic and organizational needs.

Economic evaluations conducted in chronic disease settings suggested that telemedicine is associated with cost savings, mostly indirect costs, primarily driven by reductions in travel expenses, time off work, and productivity loss for patients and caregivers [13,14]. Several studies have evaluated the economic impact of telemedicine compared with traditional in-person care [17,18,19]. An Italian national evaluation found a median per-visit savings of approximately €97 for telemedicine compared to in-person visits, largely driven by eliminated travel and associated costs for patients and caregivers [20]. A retrospective study by Waibel et al. evaluated the first 2 years of a synchronous patient-to-allergist TeleAllergy platform. The authors demonstrated significant indirect cost savings, with 200 workdays or school days saved and $58,000 in travel-related costs and 80,000 km in driving avoided [21].

**Figure 1:**
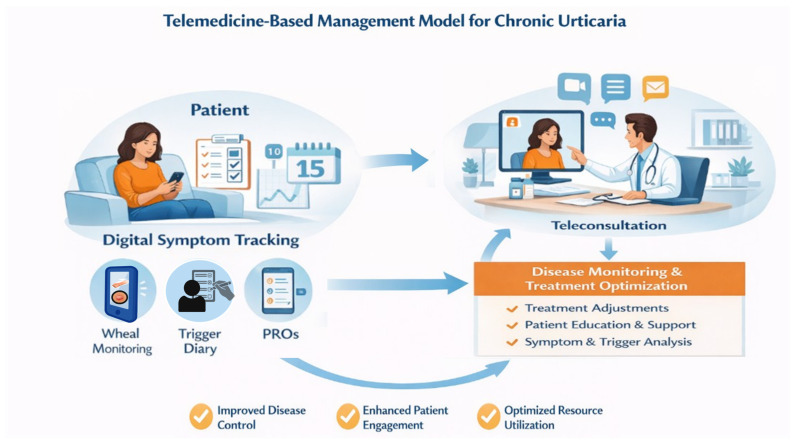
Telemedicine-Based Management Model for Chronic Urticaria (a conceptual framework). Created in BioRender. Inchingolo, R. (2026) https://BioRender.com/0ns9b9d, (accessed on 22 March 2026).

The EAACI position paper on “Telemedicine with special focus on allergic diseases and asthma” underline that the long-term effects of telemedicine on healthcare costs and patient outcomes are not currently precisely established [22]. In fact, we lack clear data on implementation costs (platforms, staff training, workflow redesign, regulatory compliance). Recently, Eze et al. systematically reviewed published data on benefits of telemedicine highlighting the need to improve generalizability and reporting standards [23]. The most common barriers to implementation were usability and lack of reimbursement [23]. Although disease-specific cost-effectiveness data for chronic urticaria remain limited, the available evidence suggests that telemedicine may represent a cost-saving or cost-neutral complementary care model, particularly in conditions requiring frequent follow-up and long-term monitoring. From a healthcare system perspective, telemedicine may also improve resource allocation by optimizing visit scheduling, reducing unnecessary outpatient consultations, and facilitating earlier therapeutic adjustments based on remotely collected data (Figure 1). Nevertheless, further high-quality, disease-specific economic evaluations are needed to fully quantify the cost-effectiveness of telemedicine-based management strategies in chronic urticaria.

## 3. Aim and Scope

The primary aim of this scoping review is to critically examine the role of telemedicine and digital health interventions in the management of urticaria, with particular emphasis on their potential to address the clinical and organizational challenges. The review briefly summarized current knowledge of epidemiology, pathophysiology, clinical presentation, and standard therapeutic approaches to urticaria.

Specifically, this review aims to evaluate how telemedicine-based strategies—such as teleconsultations, mobile health applications, digital symptom diaries, and electronic patient-reported outcome measures—can support trigger identification and long-term treatment adherence in patients with acute and chronic urticaria. Furthermore, this review seeks to explore the impact of digital health solutions on patient empowerment, shared decision-making, quality of life, and healthcare resource utilization. By highlighting current evidence, practical applications, and existing gaps, this review aims to provide a framework for the integration of telemedicine into routine clinical practice and to identify future directions for research and innovation in the digital management of urticaria.

## 4. Methods

In this scoping review, we conducted a literature search for published articles on “Urticaria” and “Telemedicine” from 1 January 1997 to 30 January 2026 adopting the international PubMed/Medline library. The research utilized the following query: ((“urticaria”[MeSH Major Topic]) OR (urticaria[Title/Abstract]) OR (acute spontaneous urticaria[Title/Abstract]) OR (chronic spontaneous urticaria[Title/Abstract]) OR (inducible urticaria[Title/Abstract]) OR (solar urticaria[Title/Abstract]) OR (cold urticaria[Title/Abstract]) OR (heat urticaria[Title/Abstract]) OR (syntomatic dermgraphism[Title/Abstract]) OR (delayed pressure urticaria[Title/Abstract]) OR (contact urticaria[Title/Abstract])) AND ((telemedicine[Title/Abstract]) OR (smarthphone[Title/Abstract]) OR (mobile[Title/Abstract]) OR (store-and-foward[Title/Abstract]) OR (asynchronous[Title/Abstract]) OR (virtual[Title/Abstract])) NOT ((systematic review[Filter]) OR (guideline[Filter]) OR (meta-analysis[Filter]) OR (practice guideline[Filter]) OR (scoping review[Filter])) AND (english[Filter]) AND (1997:2026[pdat]).

Studies were selected based on following inclusion criteria: (1) human studies, (2) adaptive clinical trial, randomized controlled trials, observational studies, pragmatic clinical trial, or case–control studies, (3) studies involving telemedicine-based strategies in patients affected by urticaria with assessed data and available abstract. Ineligible records included other sources such reviews, meta-analysis, position paper, books chapter, editorials, scientific clinical conference, and anthologies from research institutes editorials, non-human research, studies without incomplete clinical outcome data, and those with only an abstract in English but a full text in a language other than English. Studies that reported mixed “teledermatology” cohorts were included only when urticaria-specific results were extractable or verifiably incorporated into the endpoint. Studies on artificial intelligence (AI) were excluded. Selected papers were manually and individually verified by two authors (AR and ET) and duplicates manually removed.

## 5. Results

**Figure 2:**
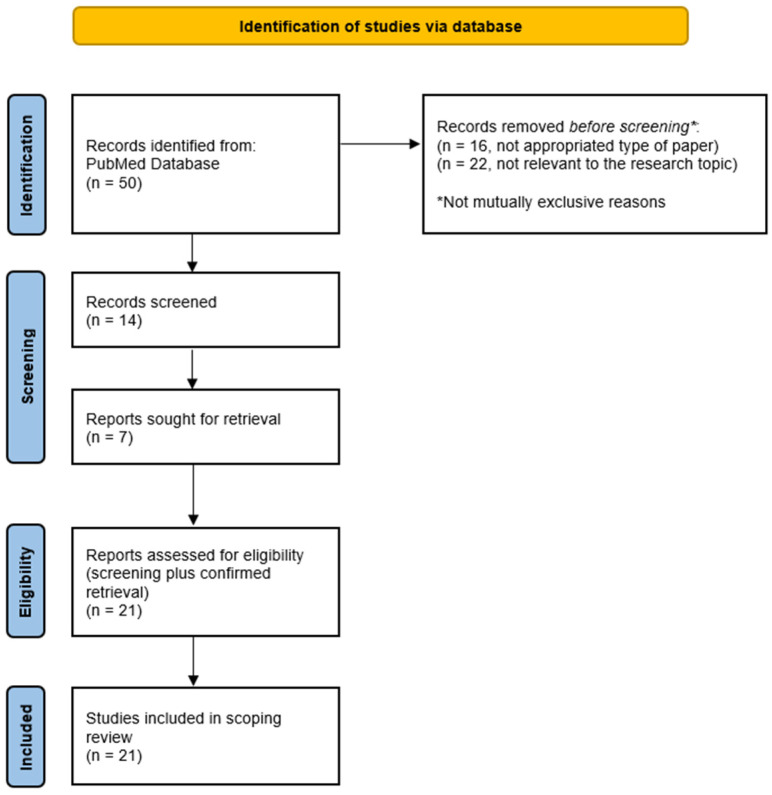
Flowchart of research strategy.

The research strategy allowed us to identify 50 papers [24,25,26,27,28,29,30,31,32,33,34,35,36,37,38,39,40,41,42,43,44,45,46,47,48,49,50,51,52,53,54,55,56,57,58,59,60,61,62,63,64,65,66,67,68,69,70,71,72,73]. Sixteen papers [29,33,36,41,43,47,48,49,50,52,53,54,59,61,70,71] were excluded from the selection process because of type of paper (review, position paper or comment). Twenty-two papers were excluded because focused on different topic [24,28,30,34,35,36,38,39,44,46,49,56,60,62,65,68,69,72,73] or teledermatology [25,55,58]. Seven papers [74,75,76,77,78,79,80] were manually and individually added to the list by AR and ET. Therefore, twenty one papers were selected [26,27,31,32,37,40,42,45,51,57,63,64,66,67,74,75,76,77,78,79,80] [Table 1]. Figure 2 described PRISMA flow-chart of the research strategy.

## 6. Telemedicine in Urticaria

### 6.1. Definition and Modalities

The official definition of telemedicine is provided by the World Health Organization, dates to 1997, and defines telemedicine as a tool for delivering of appropriate medical care to patients who are physically distant through the combined use of information technology and telecommunications [81]. The term telemedicine comprises two distinct elements: telecommunication technology, that is, the use of telematic tools; medicine, that is, the application of these tools for telemedical purposes.

Telemedicine is therefore a combination of telecommunications and medicine, where the former is used to support the latter. It is considered an evolution of traditional medicine at a digital level, ready to provide tools that enable remote communication between doctors through different methods such as text, sound, or images, supporting prevention, diagnosis, treatment, and monitoring [82].

Telemedicine is divided into different categories, depending on the purpose for which it is used [83]. Teleconsultation is a form of telematic communication that allows doctors to interact remotely with their colleagues, exchanging opinions on specific cases to improve the clinical decision-making process and allows doctors to interact remotely with their patients. This form of communication does not require the physical presence of the patient and can be used in real-time (synchronous mode) typically via video or telephone consultations or through an exchange of documents or information (asynchronous mode or “store-and-forward”) [84]. It is useful for obtaining a second opinion, to improve the delivery and quality of care. Remote patient monitoring is focused on maintaining continuity even remotely, particularly for chronic patients or those in rehabilitation. Using specific devices, vital data such as blood pressure, or important clinical data such as images of skin lesions, or laboratory results, and patient-reported outcomes are collected. The data is immediately sent to healthcare personnel so that they can act if significant changes are observed. Telemonitoring is particularly useful for patients who frequently undergo check-up visits. In the context of urticaria, these approaches play an important role in ensuring clinical follow-up, symptom monitoring, and therapeutic adjustment [85]. Hybrid models can integrate these approaches, combining remote consultations and face-to-face visits, especially in cases that require more complex management or in-depth diagnostic analysis [86,87].

In this context, instant messaging (one-to-one platforms like WhatsApp) represents the most frequent ICT (Information and Communication Technologies) modality in the daily lives of urticaria patients. The UCARE (Urticaria Centers of Reference and Excellence) study revealed that 85.4% of subjects use it daily [80]. This finding highlights the potential of such tools for rapid and direct contact, facilitating the immediate transmission of queries or clinical images. In clinical practice, this is often defined as an “informal telemedicine” tool, acting either synchronously (if the physician responds immediately via chat/video call) or asynchronously (if the patient sends data for later review by the physician) [80].

A practical example of teledermatology is the MindMySkin platform, used for managing patients with atopic dermatitis, eczema, psoriasis, and chronic urticaria. This platform not only allows us to monitor cutaneous signs but also provides psychological support tools and real-time symptom management strategies, enhancing patient self-management. Consequently, modern teledermatology no longer consists solely of sending clinical images for remote evaluation but is evolving toward a 360-degree digital patient management model [64].

### 6.2. Clinical Applications of Telemedicine in Urticaria

The chronic and fluctuating nature of urticaria, coupled with the need for frequent follow-ups, makes these modalities particularly suitable for monitoring therapeutic efficacy, systematically collecting patient-reported outcomes, and supporting personalized care pathways. Telemedicine has been progressively integrated into various stages of chronic urticaria management.

Specifically, it can serve as a pre-diagnostic clinical screening tool, useful for prioritizing specialist referrals and identifying clinical scenarios that require in-person evaluation without replacing the traditional diagnostic process [57,88]. Furthermore, telemedicine is well-suited for monitoring disease activity and clinical control through the systematic use of validated patient-reported outcome measures, such as Urticaria Activity Score (UAS) [52,89,90], Chronic Urticaria Quality of Life Questionnaire (CU-Q2oL) [91], and Urticaria Control Test (UCT) [91,92], often integrated into digital diaries or remote monitoring systems.

More specifically, it is used for therapeutic follow-up, especially in patients undergoing second- or third-line therapies, where dosage adjustments or treatment switches may be necessary. This allows for closer monitoring and facilitates timely therapeutic interventions [27,88]. A concrete example of a mobile health (mHealth) application in chronic urticaria management is the CRUSE^®^ (Chronic Urticaria Self Evaluation) app, developed within the UCARE (Urticaria Centres of Reference and Excellence) network. This platform allows patients to independently record UAS7 and UCT data over time, document medication use, and upload images of skin lesions. Preliminary real-world data from international app usage suggests high acceptability, supporting the role of mHealth applications as complementary tools to telemedicine for disease monitoring and active patient engagement [42].

Finally, it provides valuable support for patient education, including guidance on avoiding triggers, correct antihistamine use, and managing flare-ups, thereby fostering active patient involvement and self-management [57,88]. Beyond active monitoring via photos and questionnaires, emerging studies propose using smartphones for passive monitoring.

### 6.3. Use of Social Media

Some observational studies have employed digital tools for patient recruitment, such as closed Facebook groups dedicated to chronic urticaria, where questionnaires for patient characterization were provided [77]. This type of recruitment has proven particularly useful for rare forms of urticaria, enabling the acquisition of an international, multicentric sample without requiring physical access to specialized center. A relevant example is solar urticaria (<0.5% of all CU), for which Facebook recruitment allowed for the enrollment of 112 patients—a significant number for a rare pathology [74].

Beyond recruitment, social media can be utilized in numerous ways, becoming an effective clinician’s tool for patient education and improving treatment outcomes. Facebook and Instagram can be used to disseminate accurate information and counter “fake news”; LinkedIn can serve as a space for professional interaction and staying updated on the latest guidelines by sharing scientific articles and announcing new research findings. Furthermore, monitoring social media allows clinicians to understand patient “attitude”—identifying the most frequent doubts and the symptoms that cause the most concern—thereby improving communication during in-person visits [76].

The effectiveness of social media, as evidenced by patient recruitment via closed Facebook groups [74,77], is supported by UCARE study data [80]. Facebook is used by 13.6% of patients for urticaria-related information, with a significantly higher prevalence in rural areas (26.2%) compared to urban ones. However, literature highlights a paradox: despite its use for recruitment and peer exchange, patients attribute lower qualitative value to Facebook compared to search engines or institutional websites. This confirms that while social media platforms are powerful tools for connecting patients (especially for rare forms like solar urticaria), they are not yet considered authoritative sources for the clinical management of the pathology [80].

## 7. Evolution of Telemedicine: Pre-COVID and Post-COVID

Before the COVID-19 pandemic, the use of telemedicine in the management of urticaria was limited and largely confined to selected referral centers or experimental settings. Remote care was mainly applied to follow-up visits in patients with chronic spontaneous urticaria (CSU) with a well-established diagnosis and treatment plan, while initial diagnosis, therapeutic escalation, and biological initiation were almost exclusively conducted through in-person consultations [93,94]. Regulatory constraints, lack of reimbursement, and limited digital infrastructure represented major barriers to widespread implementation. As a result, despite the fluctuating nature of urticaria and the need for frequent monitoring, telemedicine played only a marginal role in routine clinical practice [94].

The COVID-19 pandemic marked a turning point in the adoption of telemedicine for urticaria care. Restrictions on non-urgent outpatient visits and the need to protect vulnerable patients accelerated the integration of teleconsultations and digital health tools into daily practice [95]. In CSU, telemedicine proved particularly effective in ensuring continuity of care, allowing remote assessment of disease activity, monitoring of treatment response, and timely therapeutic adjustments, including patients receiving biologic therapies such as omalizumab [95,96]. The pandemic also fostered the use of digital symptom diaries and patient-reported outcome measures (e.g., UAS7), enabling structured and longitudinal disease monitoring [91]. Post-COVID, telemedicine is increasingly recognized as a stable and complementary component of urticaria management, particularly for long-term follow-up, elderly or comorbid patients, and those living in remote areas, while in-person visits remain essential for diagnostic confirmation and complex clinical decision-making [94,97].

## 8. Telemonitoring of Biologic Therapies

Telemonitoring represents a particularly valuable strategy in patients with chronic urticaria treated with biologic therapies, especially omalizumab, which is often administered over prolonged periods and requires regular assessment of efficacy and safety [9,98]. Digital health tools, including electronic symptom diaries and mobile applications, allow continuous monitoring of disease activity, treatment adherence, and quality of life outside the clinical setting [91,97]. This approach enables early identification of suboptimal response, loss of disease control, or relapse after treatment interruption, facilitating timely therapeutic adjustments without the need for frequent in-person visits [97].

In addition to clinical monitoring, telemedicine supports patient education and empowerment in biologic therapy management. Remote consultations can be used to reinforce adherence, address concerns regarding treatment duration or adverse events, and provide guidance on self-administration when applicable [86]. Telemonitoring is particularly beneficial for elderly patients or those with multiple comorbidities, for whom repeated hospital visits may be challenging, and for patients followed at tertiary centers but residing far from referral hospitals [86].

## 9. Opportunities and Benefits

Although the diagnosis of urticaria is essentially clinical and direct inspection cannot always be fully replaced by indirect observation through photos or videos, telemedicine can play a valuable role as a triage and follow-up tool, helping to reduce unnecessary access to emergency services. Structured remote interviews conducted via video or phone calls or online questionnaires—focusing on symptom characteristics, affected body areas, and the duration and progression of lesions—may support first-line symptomatic management and identify patients who require in-person evaluation. This approach is particularly relevant in acute urticaria, where outpatient visits are frequently requested even after treatment has already been initiated in the emergency department (ED). In the hours or days following ED discharge, symptoms may rebound despite appropriate acute care, often causing significant patient anxiety and leading to repeated ED visits or urgent allergy consultations, sometimes at personal expense. In these circumstances, access to teleconsultations may allow close monitoring of symptom evolution, guidance on continuation or adjustment of home therapies (e.g., antihistamines and short courses of oral glucocorticoids when appropriate), reassurance regarding the typically benign course of the condition, and clarification of the appropriate timing for allergy investigations when an allergic trigger is suspected (e.g., drugs or foods). As diagnostic tests are generally uninformative or contraindicated during the acute phase, telemedicine may help prevent premature testing and reduce avoidable healthcare costs.

Guideline-based treatment—second-generation antihistamines, including dose escalation up to fourfold when indicated, followed by anti-IgE biologics in non-responsive cases—often, but not invariably, achieves adequate symptom control. In this setting, telemedicine is particularly useful during follow-up of patients with an established diagnosis and treatment plan. In fact it facilitates early identification of disease flares requiring temporary treatment escalation, monitoring therapeutic effectiveness, and supporting shared decisions regarding continuation or discontinuation of therapy. Over prolonged treatment courses (e.g., 12 months), replacing several in-person visits at referral centers with remote consultations may result in meaningful savings in travel costs and work time for patients, while also improving clinical workflow efficiency. These advantages are especially relevant for elderly patients, individuals with multiple comorbidities (e.g., oncologic or metabolic diseases), and those living in remote geographical areas who may face difficulties accessing hospital-based care.

Several potential benefits of telemedicine in chronic urticaria management have been reported, including improved access to specialist care for patients in underserved areas, reduced waiting times and decreased need for face-to-face visits, although the strength of evidence remains heterogeneous and partly derived from non-urticaria-specific settings, unnecessary face-to-face visits. Digital tools such as mobile applications and electronic diaries further support standardized symptom monitoring through quality-of-life questionnaires enable timely recording of itching, wheals, and angioedema, and facilitate medication reminders and adherence tracking (Table 2). Importantly, this longitudinal data can be reviewed by clinicians even before scheduled visits, allowing more targeted and timely therapeutic adjustments. Finally, telemedicine may also serve an important educational role, including training in subcutaneous self-administration, management of local injection-site reactions, and re-training of correct techniques when therapies are resumed after prolonged intervals. High levels of patient satisfaction have consistently been reported, largely due to increased convenience and the perception of improved continuity of care [26,27,57].

## 10. Limitation and Risks

Despite the multiple advantages of telemedicine for urticaria management and the encouraging experience gained in other allergic and non-allergic diseases; several limitations must be carefully considered. From a clinical perspective, telemedicine is most appropriately applied in well-defined scenarios, particularly in the follow-up of patients with an established diagnosis of chronic urticaria, monitoring of disease activity, and therapeutic adjustments in clinically stable patients. It may also represent a useful tool for post-emergency department follow-up in acute urticaria and for patient education and adherence support. Conversely, urticaria diagnosis remains largely dependent on direct clinical inspection, and in-person evaluation is essential in several situations. These include initial diagnostic assessment, significant diagnostic uncertainty, atypical skin lesions (e.g., lesions persisting >24 h, painful or burning rather than pruritic, or leaving residual purpura), suspected urticarial vasculitis, isolated angioedema, or any clinical suspicion of anaphylaxis. Although video tools have substantially improved, reliance on indirect observation through photos or video may entail a residual risk of diagnostic error, and it may delay recognition of early urticaria occurring in the context of evolving anaphylaxis, a scenario that requires immediate in-person medical intervention. For this reason, telemedicine should be embedded within a structured safety framework that includes predefined triage criteria and clear thresholds for conversion to face-to-face evaluation. In general, remote assessment should be limited to clinically stable patients and follow-up visits, while in-person assessment is strongly recommended in the presence of significant diagnostic uncertainty and/or urticaria in the context of other pathologies (e.g., anaphylaxis). In addition, clinicians should ensure that patients receive explicit instructions on warning symptoms requiring urgent medical attention and on how to access emergency care. In this regard, regulations and professional medical liability insurance frameworks should explicitly address the risks associated with remote assessments, including the possibility of misclassification or delayed escalation to urgent care.

Additional constraints relate to the inability to perform a complete physical examination, which is particularly relevant during initial evaluation and differential diagnosis (e.g., urticarial vasculitis, isolated angioedema, or other dermatoses) [42]. Moreover, variability in smartphone camera quality and lack of standardization may limit the reliability of visual data for clinical decision-making.

To give practical examples, an in-person evaluation should be strongly recommended or mandatory when a patient reports signs or symptoms of possible anaphylaxis, which must always be investigated by the physician with targeted questions. It is mandatory to ask the patient if, in addition to the skin condition, they experience symptoms such as dyspnea, wheezing or chest tightness, dysphonia or difficulty swallowing, dizziness, fainting, acute gastrointestinal symptoms (e.g., vomiting, diarrhea), or rapidly progressive angioedema. An affirmative answer to any of the above questions is a criterion for referring the patient to emergencies/urgent services. Furthermore, an in-person visit is indicated if the patient reports angioedema without wheals and/or itching, almost exclusive involvement of the lips, tongue, palate, or larynx (hoarseness/dysphonia/stridor), previous episodes of isolated, unexplained angioedema, and/or a family history of angioedema. In this case, the suspicion of bradykininergic angioedema requires direct evaluation, to allow examination of the airways and administer appropriate therapy in case of pharyngolaryngeal edema. If needed the specialist will advise the patient to perform laboratory tests (e.g., complement C4 fraction, quantitative and functional C1 inhibitor and genetic tests) for the correct differential diagnosis of angioedema. The televisit should be converted to an outpatient visit if indirect observation and/or triage questions reveal atypical skin lesions, such as those that persist in the same location for >24 h, are painful or burning rather than itchy, leave residual purpura or hyperpigmentation, or are associated with fever and/or other systemic symptoms. Suspected urticaria-vasculitis and/or skin lesions appearing in the context of autoimmune diseases/systemic connective tissue diseases require a clinical, laboratory, and instrumental evaluation. Generally, an in-person visit is preferable for the initial assessment of a patient with a history suggestive of chronic spontaneous urticaria who, however, is naive to previous screening tests and/or in-person dermato-allergy examinations. In all cases where a teleconsultation is performed, during which the image quality is poor/inadequate and the diagnosis is therefore uncertain, a supplementary in-person visit, even if deferrable, is preferable. If, during patient follow-up, a lack of response to first-line therapy or an unexpected loss of response/worsening of symptoms is noted, a direct clinical evaluation of the patient is recommended before implementing a therapeutic step-up (e.g., introduction of biologics or immunosuppressants). Finally, the patient must always be guaranteed access to an outpatient visit to the referral center in cases where, due to unforeseeable technical issues (e.g., internet connection) or subjective difficulties in using digital platforms, the televisit is not conducted and/or completed adequately.

When telemedicine is implemented on a scale, practical and economic aspects become crucial. Initial costs, workflow integration, and governance models should be evaluated in advance, including the choice of dedicated, institutionally licensed platforms; whether teleconsultations can be conducted from any location using the clinician’s own IT equipment; or whether they should be restricted to hospital-based hardware and networks. Platform selection should prioritize interoperability with clinical records, structured longitudinal data capture, and clear documentation pathways. At a minimum, teleconsultations should include documentation of the modality of the visit (video or asynchronous review), patient consent, the clinical information and images reviewed, the diagnostic assessment, therapeutic decisions, and any recommendation for in-person reassessment or urgent referral. Data protection and privacy are also central issues, particularly when patients upload sensitive images of skin lesions prior to consultations. The use of non-healthcare platforms raises additional concerns: for example, although WhatsApp is widely used among urticaria patients (as reported by UCARE), it is not certified as medical software (SaMD), does not support standardized longitudinal data collection, and tends to fragment information within chats rather than integrating it into the patient’s medical record [80].

Equity considerations further limit applicability. Inequalities in access to digital technologies and digital literacy may disproportionately affect elderly patients and socioeconomically disadvantaged groups [42,57]. Importantly, barriers are not purely technical: having a device is insufficient if patients lack the skills to use platforms effectively. The UCARE study highlights a direct association between educational level and ICT use, suggesting that telemedicine tools should be tailored to patients’ socio-cultural background to avoid widening healthcare disparities [99]. Generational effects also matter while one-to-one messaging tools may be used across age groups, the use of more complex platforms declines markedly in patients over 40 [99]. Therefore, telemedicine programs should include technical support, simplified user pathways, and contingency plans (e.g., rapid switch to phone consultation or expedited in-person assessment) to address connection problems—particularly relevant for elderly individuals or patients living in areas with limited internet coverage.

Finally, methodological concerns should be acknowledged when digital platforms are used for research or patient engagement. Recruitment via social media groups may introduce selection bias, as such communities often attract individuals with more severe symptoms or dissatisfaction, and may also lead to gender participation bias. In addition, self-reported diagnoses may not be formally validated, increasing the risk of misclassification and affecting estimates of prevalence, severity, and treatment response [74]. Taken together, these limitations underscore the need for robust clinical pathways, certified and secure platforms, equitable implementation strategies, and stronger evidence from well-designed studies to support widespread adoption of telemedicine in urticaria care.

Beyond technological and organizational aspects, telemedicine in urticaria management raises important concerns related to clinician workload, medicolegal liability, and patient safety that warrant explicit discussion.

Although telemedicine is often promoted as a time-saving strategy, evidence suggests that remote care may increase administrative workload for clinicians, particularly due to asynchronous messaging, documentation requirements, image review, and integration of patient-reported outcomes into electronic medical records [83,100]. In chronic spontaneous urticaria (CSU), where disease activity fluctuates and patients frequently seek reassurance during flares, unrestricted digital access may further increase clinician burden if not supported by structured triage systems and predefined communication pathways.

Medicolegal liability represents another critical issue. Remote assessment inherently limits physical examination and may complicate differential diagnosis with other conditions. In acute urticaria with potential progression toward anaphylaxis, delayed recognition of red-flag symptoms via teleconsultation may carry significant clinical consequences. Clear documentation standards, triage algorithms, and insurance coverage adapted to remote clinical decision-making are therefore essential components of safe implementation.

Patient safety incidents and diagnostic discordance have been described in teledermatology, often related to suboptimal image quality, incomplete clinical information, or overreliance on patient-reported descriptions [101]. In urticaria management, particularly in biologic-treated patients, future research should systematically evaluate adverse events, near-misses, and safety outcomes to better define the limits of safe remote care.

## 11. Future Directions

Both patients and clinicians have emphasized that telemedicine is not universally interchangeable with in-person care. While initially adopted to triage patients, ensure continuity of care, and reduce unnecessary face-to-face visits, telemedicine is increasingly viewed as a means of extending allergy and immunology services to rural and underserved areas, mitigating specialist shortages, and reducing the travel burden on patients.

It is important to note that pre-pandemic outcome data may not be entirely applicable to the post-COVID era, prompting a surge in research activity within allergy-related telemedicine. The advancement of future research will depend on the conduct of rigorous, multi-system studies that include the identification of disease-specific telemedicine metrics. Another key requirement will be the evaluation of optimal platforms and technologies aligned with evidence-based policy decisions to sustain long-term access and reimbursement. Digital applications incorporating symptom monitoring and predefined action plans may support telemedicine-based follow-up for patients receiving biological therapies, facilitating the management of large local or delayed hypersensitivity reactions occurring outside standard observation periods. Conversely, atopic dermatitis appears particularly well suited to app-based monitoring, as established baseline treatment plans can be remotely intensified during disease flares through structured digital guidance [102]. Preliminary telemedicine consultations have demonstrated utility in patient triage by enabling the identification of procedures that require in-person evaluation, thereby reducing unnecessary clinic visits. However, a major limitation to telemedicine implementation remains the heterogeneity of healthcare infrastructure and capacity across regions, which hinders the feasibility of remote assessments. Patients who lack adequate access to or proficiency with digital technologies must therefore continue to receive care through traditional in-person visits. The successful implementation of innovative telemedicine-based care pathways will require parallel advances in health policy and regulatory frameworks, particularly regarding reimbursement structures. Furthermore, core elements of allergy and immunology practice, including food and drug challenges and allergen immunotherapy, must be thoughtfully incorporated into telemedicine-enabled models of care. Ongoing efforts to improve platform functionality are essential to ensure broad accessibility while maintaining high standards of data security and patient confidentiality [103].

A critical priority for the future of telemedicine in urticaria management is the generation of high-quality clinical evidence that goes beyond feasibility and satisfaction metrics to robustly evaluate clinical outcomes and cost-effectiveness. While a substantial body of research has demonstrated that telemedicine can improve access to care, continuity, and patient satisfaction across chronic conditions, systematic evidence on economic impact remains limited and methodologically heterogeneous. An umbrella review of telemedicine interventions across specialties found that although a significant proportion of cost-effectiveness analyses suggested potential savings, the overall quality and generalizability of these studies were constrained by low methodological rigor and inconsistent reporting standards, highlighting a major gap in the literature that must be addressed for informed policy decisions [104,105]. Furthermore, cost-minimization analyses in chronic disease contexts have shown modest reductions in societal costs—primarily due to decreased travel and productivity losses—underscoring the importance of comprehensive economic evaluation frameworks that capture both direct and indirect costs associated with remote care delivery [106].

In the specific setting of biologic therapy for chronic urticaria, high-quality prospective studies and health economic models are particularly needed to ascertain whether telemonitoring and hybrid care models can reduce overall healthcare expenditure while maintaining or improving clinical outcomes. Such research should assess not only reductions in emergency department visits and outpatient consultations but also long-term impacts on disease control, quality of life, and healthcare resource utilization. These data will be essential to support sustainable reimbursement models and integration of telemedicine into standard care pathways for urticaria.

Future developments should aim to foster a synergistic integration of artificial intelligence, telemedicine in the management of urticaria—drawing inspiration from models already developed in teledermatology—and in-person clinical evaluation, creating complementary care pathways that enhance diagnostic accuracy, optimize patient management, and maintain high standards of clinical oversight [107].

## 12. Discussion

The present review provides an urticaria-focused overview of telemedicine applications across the continuum of care, including diagnosis, disease monitoring, biologic therapy management, and long-term follow-up. Although telemedicine has been extensively investigated in dermatology and chronic disease management, analyses specifically addressing CU remain limited. It should be noted that a significant proportion of the available evidence informing telemedicine use in urticaria is extrapolated from broader teledermatology or mixed-disease studies, with relatively few studies specifically focused on chronic urticaria. CU represents a particularly suitable condition for telemedicine integration due to its fluctuating course, the central role of patient-reported symptom monitoring, and the need for longitudinal follow-up. Unlike many dermatologic conditions, the assessment of disease activity in CU predominantly employs validated PRO measures, such as UAS7 and UCT. These instruments can be reliably collected through digital platforms, facilitating remote disease monitoring. Previous systematic reviews in teledermatology have reported clinical outcomes comparable to in-person follow-up for chronic inflammatory skin diseases, such as psoriasis and atopic dermatitis [29]. However, urticaria has often been only marginally represented in these analyses, highlighting the need for disease-specific analyses such as the present review.

Evidence from studies conducted within the UCARE network further supports the feasibility of digital symptom-monitoring platforms in patients with chronic urticaria, demonstrating high patient engagement and improved documentation of disease activity [25]. Digital symptom tracking may allow clinicians to better capture disease variability over time and to adjust therapy more promptly when disease control deteriorates. Similar benefits have been reported in the broader telemedicine literature, where remote monitoring has been associated with improved treatment adherence and clinical outcomes in several chronic conditions, although direct evidence in chronic urticaria remains limited [108,109]. Beyond clinical aspects, telemedicine may also offer relevant organizational and economic advantages. Patients with chronic urticaria frequently require repeated follow-up visits, particularly when receiving biological therapies such as omalizumab or emerging biologic agents targeting type-2 inflammatory pathways. Telemonitoring may support treatment evaluation and safety assessment and has the potential to reduce the need for frequent in-person visits, although this effect has not been consistently demonstrated in urticarial-specific studies. This approach may decrease the burden of patient travel, indirect costs, and healthcare resource utilization. Economic analyses have suggested potential cost savings, particularly when telemedicine reduces patient travel and improves healthcare resource allocation [106]. Although cost-effectiveness data specifically addressing urticaria remain scarce, evidence from other chronic dermatologic and allergic diseases suggests that hybrid care models combining remote and in-person visits may represent a scalable and cost-efficient strategy.

Digital PRO platforms may also positively influence patient experience. Previous studies in chronic inflammatory diseases have shown that digital monitoring tools can improve patient satisfaction and the perception of disease control. In the context of urticaria, these tools may be particularly valuable given the unpredictable nature of symptom fluctuations and the importance of continuous symptom assessment. Notwithstanding these encouraging findings, several important limitations must be considered. The diagnosis of urticaria remains fundamentally clinical and often requires direct visual evaluation and physical examination. Consequently, telemedicine cannot fully replace in-person assessment during the initial diagnostic phase or when atypical clinical presentations are suspected. Furthermore, the reliability of remote clinical assessment depends heavily on patient-generated data, including symptom reporting and photographic documentation of skin lesions. Variability in digital literacy, smartphone technology, and patient adherence may affect data accuracy and clinical decision-making.

Digital health inequalities remain a significant concern. Access to telemedicine may be limited among elderly patients, individuals with low digital literacy, or populations living in areas with inadequate technological infrastructure.

From a technological perspective, emerging tools such as artificial intelligence–assisted image analysis, passive environmental monitoring, and predictive digital biomarkers offer exciting opportunities for personalized urticaria management. These approaches align with the principles of precision medicine and may eventually facilitate early identification of disease exacerbations and individualized treatment adjustments. However, most of these technologies remain in early developmental stages and require rigorous clinical validation before routine implementation.

While previous reviews have addressed teledermatology broadly or telemedicine in allergy and immunology [110], few have systematically examined telemedicine applications across all phases of urticaria care. Several research priorities emerge from this analysis.

First, prospective randomized controlled trials comparing telemedicine-integrated care with conventional management are needed to evaluate clinical outcomes, quality of life, and cost-effectiveness in patients with CU. Second, the development of standardized digital outcome measures and validated telemedicine protocols specifically for urticaria, to ensure consistency and reliability in remote care. Finally, future studies should assess the long-term impact of telemedicine on healthcare utilization, including emergency department visits, hospitalizations, and biologic therapy optimization. Implementation strategies must be developed to ensure that telemedicine enhances, rather than exacerbates, healthcare accessibility.

## 13. Conclusions

The available evidence suggests that telemedicine may represent a valuable complementary approach across multiple stages of urticaria care, particularly for structured symptom monitoring, follow-up during treatment adjustment, and improving access to specialist evaluation. However, the current body of evidence remains limited, heterogeneous, and partly extrapolated from other chronic inflammatory and dermatologic conditions. Remote models facilitate structured symptom tracking, enable timely follow-up during treatment escalation, and improve access to specialist expertise.

Experience gained in dermatology and allergy/immunology—accelerated by the COVID-19 pandemic—has demonstrated the feasibility and acceptability of telemedicine for chronic inflammatory and allergic conditions, offering a transferable framework for urticaria management. Robust data on clinical outcomes, safety, and cost-effectiveness specifically in chronic urticaria are still lacking. Consequently, its role should be interpreted with caution, particularly when considering broader implementation in routine clinical practice. Telemonitoring appears promising, particularly for patients receiving advanced therapies, where continuous assessment of disease control and treatment response is crucial, although further disease-specific validation is required.

Nevertheless, telemedicine cannot fully replace face-to-face evaluation in urticaria in key clinical scenarios, including initial diagnosis or atypical presentations. Diagnostic accuracy still relies heavily on direct clinical inspection, and remote care carries inherent risks, including misclassification, technological variability, privacy concerns, and inequities related to digital literacy and access. Many innovative digital approaches remain experimental and lack validation through large, disease-specific clinical trials. Furthermore, regulatory, medico-legal, and infrastructural challenges continue to limit widespread implementation.

Taken together, telemedicine should currently be regarded not as an alternative but as an integrated extension of conventional urticaria care. Hybrid models that combine remote monitoring with targeted in-person visits appear to offer the most balanced and effective strategy. Future research should prioritize high-quality randomized studies, standardized digital outcome measures, and robust economic evaluations specifically focused on urticaria. Parallel efforts in health policy, digital education, and platform certification will be essential to ensure safe, equitable, and sustainable integration of telemedicine into routine urticaria management.

These considerations highlight the need for cautious, evidence-informed implementation of telemedicine in urticaria care. Importantly, much of the current evidence is not urticaria-specific and should therefore be interpreted with caution when applied to this condition.

## Figures and Tables

**Table 1:** Papers included in scoping review.

Study (Year) [Ref.]	Country	Study Design	Tool	Clinical Focus	Sample Size	Type of Urticaria	Main Findings	Limitations
von Kiedrowski et al., (2025) [26]	Germany	Model-based simulation study	Patient journey modeling framework	Impact of educational and digital interventions on management pathway and guideline adherence in CSU	Not applicable (simulations based on secondary data)	Chronic spontaneous urticaria	Disease recognition, reduction in diagnostic/treatment delays, increase in guideline adherence, and improved patient care	Simulation model with assumptions, reliance on secondary data, no prospective patient data, limited generalizability
Viegas et al., (2025) [27]	Multinational (UCARE network)	Observational retrospective study	CRUSE^®^ mobile health app (daily monitoring questionnaire)	Identification of predictors of adherence to an mHealth tool for monitoring chronic spontaneous urticaria	2085 patients	Chronic spontaneous urticaria	Higher adherence associated with older age, male sex, European residence, and monoclonal antibody use	Self-reported diagnosis, potential selection bias of app users, limited clinical data
Sousa-Pinto et al., (2025) [31]	Multinational	Observational validation study	Digital visual analogue scales (VAS) in the CRUSE^®^ mobile app	Validation of digital patient-reported outcome measures for monitoring disease activity and impact in CSU	5938 patients	Chronic spontaneous urticaria	Very high intra-rater reliability (ICC > 0.95), and moderate-high test–retest reliability and responsiveness	App-based real-world data with self-reported disease, potential selection bias of digital tool users, limited clinical verification, observational design
Pereverzina et al. (2023) [37]	Russia	Cross-sectional online survey	Patient questionnaires during COVID-19	Impact of pandemic restrictions on urticaria disease control and healthcare access	111 patients	Chronic spontaneous urticaria	Reduced access to specialists and treatment interruptions were reported	Small sample size; self-reported outcomes; pandemic-specific context
Neisinger et al. (2024) [42]	Multinational (UCARE network)	Observational descriptive study	CRUSE^®^ mobile health application	Digital monitoring and management of chronic spontaneous urticaria	2540 app users	Chronic spontaneous urticaria	App enabled recording of symptoms, disease activity and treatments; demonstrated feasibility of digital monitoring	Self-reported data; observational design; no clinical outcome comparison
Hindelang et al. (2025) [57]	Germany	Prospective pilot study	Telemedicine platforms	Acceptability and utilization of a digital health model for CSU	24 patients	Chronic spontaneous urticaria	Disease control remained stable while quality of life improved. All physicians found the digital application reliable and timesaving	Small sample size; pilot study
Edwards et al. (2026) [63]	United States (Spanish-speaking)	Pilot feasibility study	WhatsApp educational intervention	Digital education for self-management in CSU	30 patients	Chronic spontaneous urticaria	WhatsApp-based educational program was feasible and well accepted among Spanish-speaking CSU patients	Small pilot sample; short follow-up; no clinical outcome comparison
Ali et al. (2024) [75]	Denmark	Cross-sectional observational survey	Patient-taken smartphone photographs	Clinical usefulness of photographs taken by CU patients for dermatologic evaluation	148 patients	Chronic urticaria	Patient-taken photos can support remote assessment and clinical diagnosis of CU	Observational design, survey-based without longitudinal outcomes, image quality variability
Cherrez-Ojeda et al. (2021) [67]	Multinational (UCARE network)	Cross-sectional observational survey	23-item questionnaire from the UCARE CURICT study assessing patient interest in urticaria monitoring apps	Patient interest and preferences regarding use of mobile apps to monitor chronic urticaria disease activity and control	1841 patients	Chronic urticaria (CSU and CIndU)	Over half of patients were interested in using apps to monitor disease activity and control; interest was higher in females and those with both types of urticaria versus CSU alone	Self-reported data; hypothetical acceptance
Cherrez-Ojeda et al. (2021) [78]	Multinational (UCARE network)	Cross-sectional survey	23-item questionnaire on Information and communication technologies (ICT)	Interest of CU patients in ICT tools for healthcare	1841 patients	Chronic urticaria	High interest among patients in using smartphones and digital tools for disease management	Self-reported data, variation in ICT access across regions, potential selection bias of participants at UCARE centers
Zysk & Trzeciak (2023) [77]	Poland	Cross-sectional online survey	Online questionnaire for members of Facebook urticaria group	Characterization of chronic urticaria clinical features and self-reported comorbidities in affected patients	102 respondents	Chronic urticaria	Web-based survey identified disease burden and common comorbidities	Self-reported data; selection bias; predominance of female participants
Maurer et al. (2020) [80]	Multinational (UCARE network)	Cross-sectional survey	23-item questionnaire on Information and communication technologies (ICT)	Usage frequency, quality, and relevance of ICTs for health and chronic urticaria information among patients	1841 patients	Chronic urticaria	ICT use is extremely high; web browsers and messaging platforms are most frequently used for health and CU-specific information	Self-reported data; cross-sectional design
Mondal et al. (2024) [76]	India	Cross-sectional data audit	Facebook, LinkedIn and Twitter analysis	Analysis of patients’ knowledge, attitudes, and practices toward urticaria medication treatment as reflected on social media platforms	300 posts	Urticaria	Insights into patient perceptions, concerns, and misinformation about treatments, highlighting gaps in patient knowledge and potential targets for education and support interventions online	Indirect data source, potential selection bias, lack of clinical verification, inability to infer clinical outcomes from online discourse
Schielein et al. (2021) [79]	Germany	Observational survey	Online questionnaire assessing Internet addiction and healthcare utilization behaviors	Exploration of Internet addiction prevalence and its association with healthcare use	1686 patients	Psoriasis and chronic urticaria	Internet addiction as comorbidity, patient may exhibit problematic online health information-seeking behaviors, potentially impacting patient-physician interactions and healthcare utilization	Self-reported data, potential selection bias, lack of clinical verification of urticaria diagnoses
Kiefer et al. (2025) [74]	Multinational	Cross-sectional patient survey	Disease-specific Facebook group, Online questionnaire	Treatment patterns, patient-reported disease burden, and perceived effectiveness of therapies, QoL assessment	112 patients	Solar urticaria	Survey identified treatment strategies, data on disease severity, quality of life and highlighted unmet therapeutic needs	Self-reported data, diagnosis not uniformly confirmed, selection bias
Ozturk et al., (2021) [40]	Turkey	Cross-sectional survey study	Telemedicine during COVID-19	Changes in allergy practice and patient management during the COVID-19 pandemic	183 allergists	Allergic diseases including urticaria	Significant shift toward remote consultations and telemedicine	Pandemic-specific context
Choi et al. (2025) [66]	Singapore	Multicenter, randomized controlled, double-blind clinical trial protocol	Digital psychotherapeutic mobile application	Efficacy and process evaluation of a psychotherapeutic app to reduce symptom burden and improve quality of life in patients with chronic inflammatory skin diseases	Unknown	Dermatological diseases including urticaria	Protocol aimed to assess change in Dermatology Life Quality Index and secondary patient-reported outcomes, disease severity, treatment adherence, and implementation engagement; outcomes pending future trial results	Protocol only; results not yet available
Mu et al. (2021) [45]	China	Retrospective study	Mobile application–based tele dermatology platform	Tele dermatology service utilization during COVID-19	698 patients	Dermatological diseases including urticaria	Tele dermatology ensured continuity of dermatologic care during the pandemic	Small sample size; single-country study; the accuracy of diagnosis was not confirmed clinically; age-standardization was not performed
Lee et al. (2021) [51]	Taiwan	Observational descriptive study	Live-interactive tele dermatology program	Feasibility and clinical utility of teledermatology for dermatologic diagnosis and management in underserved rural areas	426 consultations	Dermatological diseases including urticaria	Teledermatology enabled remote diagnosis and management of multiple skin diseases improving dermatologic care access in rural regions	Single-program experience in Taiwan, lack of group control, heterogeneous dermatologic conditions
Chua et al. (2026) [64]	Singapore	Pilot study	MindMySkin app	Digital psychological intervention for dermatologic symptom burden	27 participants	Dermatological diseases including urticaria	Intervention demonstrated high acceptability and usability for self-management and psychological support	Early-phase study; mixed dermatologic diseases
Song et al., (2024) [32]	China	Comparative study using a standardized patient methodology	Telemedicine platforms	Assessment of quality of telemedicine care delivered by public vs. private online platforms	594 physician-patient interactions by 10 standardized patients	Dermatological diseases including urticaria	Private telemedicine platforms: higher quality in better checklist adherence, more accurate diagnosis, more appropriate prescriptions, more patient-centered communication	Limited clinical scenarios, results specific to Chinese telemedicine platforms, potential lack of generalizability

ICT: Information and communication technologies; VAS: digital visual analogue scales; CSU: chronic spontaneous urticaria; CIndU: chronic inducible urticaria; CRUSE: Chronic Urticaria Self Evaluation; UCARE: Urticaria Centres of Reference and Excellence.

**Table 2:** Digital health tools available for urticaria management and their clinical applications.

Tool Category	Clinical Application	Evidence Summary	Limitations
Mobile health applications (mHealth)	Remote monitoring of disease activity, symptom tracking, and treatment adherence using validated patient-reported outcome measures (e.g., UAS7, UCT)	Real-world studies demonstrate good feasibility, high patient acceptance, and improved longitudinal monitoring of disease control	Requires patient engagement and digital literacy Limited randomized controlled trials
Teleconsultation platforms	Remote clinical follow-up, treatment adjustment, and specialist consultation	Demonstrated clinical utility for follow-up of chronic inflammatory skin diseases, including urticaria	Requires appropriate infrastructure and regulatory compliance Limited role in initial diagnosis
Secure messaging tools	Rapid patient–physician communication reporting of symptom changes	Improves communication efficiency, continuity of care, and clinical decision-making	Potential data privacy concerns Lack of standardization across platforms
Electronic patient-reported outcome (ePRO) systems	Standardized remote assessment of disease activity and treatment response	Validated tools allow reliable remote disease monitoring and support treatment optimization	Requires consistent patient adherence with digital tools

## Data Availability

No new data were created or analyzed in this study. Data sharing is not applicable to this article.

## Abbreviations

The following abbreviations are used in this manuscript:

AI	Artificial intelligence
CIndU	Chronic inducible urticaria
CMS	Centers for medicare and medical service
CRUSE	Chronic urticaria self-evaluation
CSU	Chronic spontaneous urticaria
CU	Chronic urticaria
CU-Q2oL	Chronic urticaria quality of life questionnaire
HAE	Hereditary angioedema
ICT	Information and communication technologies
IT	Information technology
MHealth	Mobile health
PROs	Patient-reported outcomes
saMD	Software as a medical device
UAS	Urticaria activity score
UCARE	Urticaria centers of reference and excellence
UCT	Urticaria control test
WHO	World Health Organization
